# Transverse Myelitis Associated with *Cryptococcus neoformans* in an Immunocompetent Patient

**DOI:** 10.1155/2022/2000246

**Published:** 2022-02-21

**Authors:** David B. Villafuerte, Marco F. Passeri, Rayan Qazi, Moulika Baireddy, Fernando L. Sanchez

**Affiliations:** University of the Incarnate Word School of Osteopathic Medicine, Laredo Medical Center, 1700 E Saunders St, Laredo, TX 78041, USA

## Abstract

*Cryptococcus neoformans* is a microscopic fungus that despite its pervasiveness in the environment rarely causes infection in immunocompetent patients. In immunosuppressed patients, infections involving the central nervous system (CNS) usually present as meningitis or meningoencephalitis. Cryptococcal infections are known to cause significant morbidity and mortality in immunosuppressed patients as it is difficult to eradicate even with adequate antifungal treatment. A 44-year-old Hispanic male presented to the hospital with headache, progressive urinary retention, neck and back pain, and right upper and bilateral lower extremity weakness for five days. Imaging revealed small foci in the white matter and revealed diffuse abnormal signal involving the cervical medullary junction extending up to the thoracic spine. Analysis of cerebral spinal fluid (CSF) obtained via lumbar puncture was positive for the *Streptococcus* antigen with cultures also growing *Cryptococcus neoformans*. Upon evaluation, patient was not found to be immunocompromised. This report works to highlight an atypical presentation of a cryptococcal CNS infection to raise awareness amongst clinicians hoping to prevent a delay in diagnosis of this disease given its high mortality.

## 1. Introduction

*Cryptococcus neoformans* is an infectious yeast known to grow in the environment. It infects humans via the respiratory route usually causing a transitory asymptomatic pulmonary infection. However, mainly in immunosuppressed individuals, it may enter the CNS causing neurological manifestations such as meningitis or meningoencephalitis but seldom transverse myelitis. Herein, we report a case of transverse myelitis associated with cryptococcal infection in a middle-aged immunocompetent patient.

## 2. Case Report

A 44-year-old Hispanic male presented to our hospital with progressive urinary retention, headache, posterior neck pain, back pain, and right upper and bilateral lower extremity weakness present for five days. He did not identify an immediate prior inciting event, although he reports he had a workplace head trauma without loss of consciousness two weeks prior to admission.

He denied a history of allergies, taking supplements or medications, medical history, surgical history, or family history. Social history was remarkable for alcohol consumption of six beers daily without symptoms of withdrawal when stopping and distant IV drug use with heroin sixteen years ago. He denied tobacco use. He stated he had five previous female sexual partners but none in the past three years. He worked in construction and lived independently.

**Figure 1:**
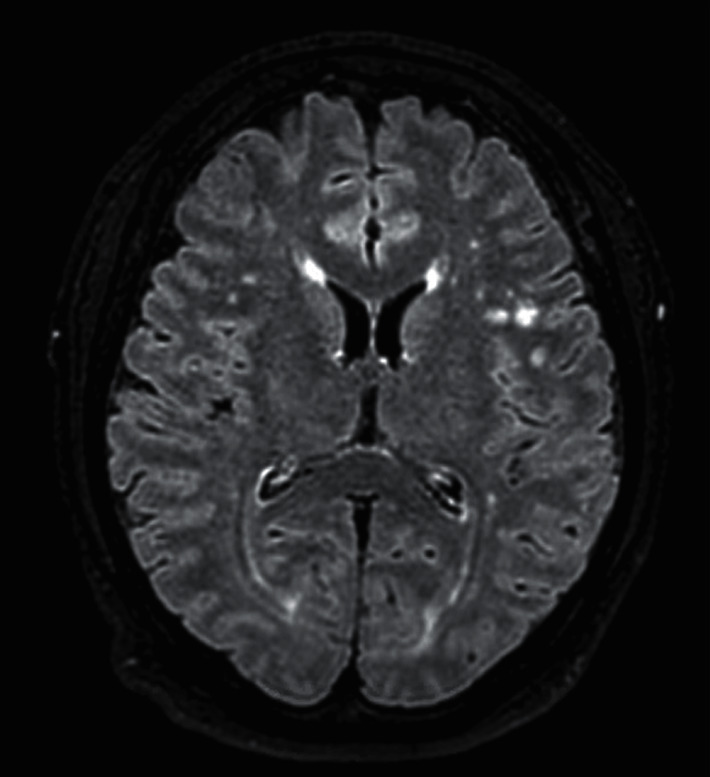
MRI brain axial FLAIR. Multifocal supratentorial ovoid and nonspecific T2 hyperintensities. Red arrow points to an ovoid T2 hyperintensity.

**Figure 2:**
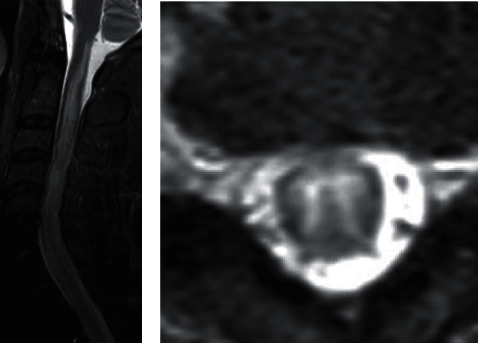
(a) MRI cervical spine sagittal STIR. Longitudinally extensive T2 hyperintense lesion with cord expansion from the cervicomedullary junction to C7. (b) MRI cervical spine axial T2. Prominent grey matter T2 hyperintensity.

On admission, vital signs consisted of a temperature of 98.9 F, heart rate of 76, blood pressure of 124/68 mmHg, and SpO_2_ of 99% with a height of 165.1 cm and weight of 95.253 kg. On initial presentation, physical exam including cardiovascular, pulmonary, and gastrointestinal systems was unremarkable. However, remarkable findings were seen in the neuromusculoskeletal system where deficits included diminished muscle strength of right lower extremity (grade 0/5), left lower extremity (grade 2/5), and right upper extremity (grade 4/5). He was experiencing fecal and urinary incontinence as well. Computed tomography (CT) without contrast of the head did not reveal intracranial abnormality. Magnetic resonance imaging (MRI) of the head revealed several small rounded foci of increased signal in the white matter in the FLAIR and T2-weighted images that were rather nonspecific, however, may be attributed to microvascular ischemic disease, vasculitis related to *Cryptococcus*, or other etiologies (Figure 1). MRI of the cervical, thoracic, and lumbar spine revealed diffuse abnormal signal with cord expansion present in the spinal cord extending from the cervical medullary junction to C7 level (Figures 2(a) and 2(b)), as well as another spinal lesion in the thoracic cord from T7-8. However, the thoracic spine lesion may have been related to spinal disc protrusion that compressed the spinal cord; which could result from inflammatory homeopathy secondary to infection (Figures 2(a) and 2(b)).

Laboratory findings were predominantly unremarkable (Table 1) except for hemoglobin, 14.00 g/dL; platelet count, 81 × 10^3^/mcL; prothrombin time (PT), 20.1 seconds; international normalized ratio (INR), 1.66 ratio; thromboplastin time partial (PTT), 43.8 seconds; aspartate aminotransferase (AST), 48 unit/L; and bilirubin total, 4.96 mg/dL. Lumbar puncture studies were remarkable for CSF glucose, 22 mg/dL and CSF protein total, <2.0 mg/dL. CSF cytology demonstrated WBC 56 cells/mm^3^, RBC 125 cells/mm^3^, neutrophils 20 cells/mm^3^, and lymphocytes 80 cells/mm^3^. CSF antigen was positive for *Cryptococcus neoformans*. Gram stain of CSF was positive with *Cryptococcus neoformans*/*gattii* complex growing from THIO broth, and mycology was positive for fungal culture revealing *Cryptococcus neoformans*. Finally, molecular testing with PCR confirmed *Cryptococcus neoformans*.

Patient was started on fourteen day induction therapy with intravenous liposomal amphotericin B at 3 mg/kg (300 mg) every 24 hours and oral flucytosine 1500 mg every 6 hours. He received daily physical therapy as well. At the end of the 14-day induction therapy, a repeat lumbar puncture was performed. Results were remarkable for CSF glucose, 33 mg/dL; CSF protein total, 126.6 mg/dL; and opening pressure, 14 cm H_2_O. Gram stain of CSF was positive for few yeast and moderate white blood cells. Therefore, induction was extended for another seven days. Third lumbar puncture was remarkable for CSF glucose, 19 mg/dL; CSF protein total, 174.4 mg/dL; and opening pressure, 14 cm H_2_O. However, gram stain revealed no microorganisms but only rare white blood cells. Induction therapy was ceased, and maintenance therapy with oral fluconazole 800 mg every 24 hours was initiated.

Upon discharge, physical exam showed muscle strength improvement with right upper extremity (grade 5/5), left lower extremity (grade 4/5), and right lower extremity (grade 3/5). However, patient continued to experience fecal and urinary incontinence. At this time, patient has scheduled follow-up with infectious disease clinic and physical therapy, but states he may move back soon to Mexico to live with family and continue medical care there.

## 3. Discussion

*Cryptococcus neoformans* and *Cryptococcus gattii* are two pathogenic yeasts that cause cryptococcosis [1]. They are found in the environment and derived from the phylum Basidiomycota. They are readily distinguished by the presence of their heavy polysaccharide capsule, which serves as its dominant virulence factor through interfering with immune responses by providing the fungal cell with an antiphagocytic defensive shield [2]. In humans, cryptococcal species penetrate via inhalation but are usually eliminated by host defense mechanisms in immunocompetent individuals. On occasion, they may progress to pneumonia or even disseminate in the CNS by crossing the blood-brain barrier [3], but this is mainly seen in immunocompromised patients. Nevertheless, it has been reported in small number of immunocompetent patients [4]. The most common clinical presentations to be associated with the CNS in both patient populations are meningitis, meningoencephalitis, cerebral parenchymal abscesses (or cryptococcomas), and hydrocephalus [5, 6]. Few cases in literature have also reported transverse myelitis due to cryptococcal infection which is the clinical presentation pertinent to our case [9–14].

To diagnose transverse myelitis, there are five inclusion criteria that need to be satisfied. First, clinical presentation of the bilateral (not necessarily symmetric) sensorimotor or autonomic spinal cord dysfunction must be present. Second, a well-defined sensory level should be established. Third, a progression to nadir of clinical deficits is usually noted between 12 hours and 21 days after symptom onset. Fourth, there is a demonstration of spinal cord inflammation, either via CSF pleocytosis, increased CSF specific oligoclonal bands, elevated IgG index (CSF IgG/serum IgG), or MRI revealing a gadolinium-enhancing cord lesion. Finally, the fifth criterion excludes compressive, postradiation, neoplastic, and vascular etiologies as a possible explanation [15]. The patient in our case satisfied four out of the five diagnostic criteria, with the exception of a well-defined sensory loss. In our case, the patient presented with bilateral motor weakness with most severe symptoms reported within the time period between 3 and 5 days. CSF pleocytosis was demonstrated with cultures yielding *Cryptococcus neoformans*. Other autoimmune and vascular workup yielded negative results. The patient did not demonstrate significant sensory loss, which can be attributed to anterior grey matter involvement in the spinal cord, which comprise the motor neurons.

Identifying the cause of transverse myelitis is essential to help predict the clinical course, to establish a treatment plan, and to reach a decision regarding whether prophylaxis to prevent a future neurological event is necessary [15]. The etiologies of transverse myelitis consist of multiple causes including various infectious or inflammatory disorders [7]. Of the infectious etiologies, the most common affecting microbes are varicella zoster, herpes simplex, cytomegalovirus, Epstein-Barr, influenza, echovirus, human immunodeficiency virus, measles, and rubella [8]. Based on our laboratory findings (Table 1), these and several other causes were excluded.

Transverse myelitis associated with *Cryptococcus neoformans* is an incredibly rare presentation. To date, there have been seven documented cases of transverse myelitis caused by Cryptococcus infection (Table 2). All these cases in literature featured patients who also were immunocompetent. Of note, surgical intervention was not pursued in our case due to lack of sizable spinal cryptococcomas on MRI [9–14].

Establishing Cryptococcus as the cause of transverse myelitis requires extensive evaluation. Diagnosis typically is made by antigen testing in the serum and/or cerebrospinal fluid, by culture, or by histopathology of infected tissues [13]. A lumbar puncture with the measurement of opening pressure is recommended for patients with suspected cryptococcosis given that elevated opening pressure is a common complication of cryptococcal meningitis that can lead to detrimental outcomes [13]. As mentioned previously, our patient was found to have both positive CSF antigen and cultures.

Typically, once CSF and culture indicate infection, it may be assumed that infection is the cause of transverse myelitis. If culture is negative, then compressive, vascular, toxic or metabolic, neurodegenerative, or neoplastic myelopathies as causes are investigated [7]. However, because of the rarity of cryptococcal origin, complete workup to evaluate the concomitant cause was pursued. As seen in imaging (Figures 1 and 2), CSF, and extensive laboratory testing (Table 1), no such concomitant etiology was present. Patient also displayed marked improvement on antifungal therapy. Therefore, cryptococcal infection is the most plausible explanation.

In sum, we report a rare case of transverse myelitis caused by *Cryptococcus neoformans* in an immunocompetent patient. As more cases are reported, early diagnosis and treatment may be possible which will minimize extent of disability and maximize recovery for these patients.

## Figures and Tables

**Table 1:** Summary of negative diagnostic tests done in our patient to rule out alternative causes of transverse myelitis.

Sputum and serologic tests	Cerebrospinal fluid tests

*∗*CBC	WNV IgG/IgM
*∗*CMP	HIV 1 and 2 Ab/Ag
UA	HBV core Ab IgM
UDS	HBV surface Ag
CRP	HAV Ab IgM
Vitamin B12 level	*Escherichia coli* K1 PCR
ESR	*Haemophilus influenzae* PCR
ANA	*Listeria monocytogenes* PCR
Antiphospholipid antibody	*Neisseria meningitidis* PCR
Ds-DNA ab	*Streptococcus pneumoniae* PCR
IgG level	HSV 1 PCR
IgG and IgM Lyme disease Ab	HSV 2 PCR
RPR	HSV 6 PCR
VDRL	VZV PCR
Aquaporin 4 ab	
**AFB**	
^*∗∗*^HCV Ab positive, PCR negative	COVID-19 PCR
	Oligoclonal bands
	Myelin basic protein
^*∗∗*^QuantiFERON-TB gold, TB PCR sputum negative	

*∗*Some abnormalities as stated in text. ^*∗∗*^Screening testing is positive, confirmatory testing is negative. Ab, antibody; Ag, antigen; AFB, acid fast bacilli; PCR, polymerase chain reaction; CBC, complete blood count; CMP, complete metabolic panel; UA, urinalysis; UDS, urine drug screen; CRP, C-reactive protein; ESR, erythrocyte sedimentation level; ANA, antinuclear antibody screen; ds-DNA Ab, double-stranded DNA antibody; IgG, immunoglobulin G; IgM, immunoglobulin M; IgG and IgM Lyme disease Ab, IgG and IgM Lyme disease antibody; RPR, rapid plasma reagin; VDRL, Venereal Disease Research Laboratory; HCV, hepatitis C virus; HBV, hepatitis B virus; HAV, hepatitis A virus; HSV, herpes simplex virus; VZV, varicella zoster virus; WNV, West Nile virus; HIV, human immunodeficiency virus.

**Table 2:** Case comparison.

First author	Villafuerte	Qu	Gultasli	Su	Grosse	Ramamurthi	Skultety	Lai

Country	USA	China	Turkey	Taiwan	Germany	India	USA	Taiwan
Age	44	55	47	Not reported	24	17	60	60
Sex	Male	Male	Male	Not reported	Female	Female	Male	Male
Back pain	Yes	No	No	Not reported	Yes	Yes	Yes	Not reported
LE weakness	Yes	Yes	Yes	Not reported	Yes	Yes	Yes	Yes
Urinary disorder	Yes	Yes	No	Not reported	Yes	No	Yes	Not reported
LE numbness	Yes	Yes	Yes	Not reported	Yes	No	Yes	Not reported
CSF glucose	Low	Normal	Not reported	Not reported	Normal	Normal	Unknown	Not reported
CSF cytology	Lymphocyte	Monocyte	Not reported	Not reported	No pleocytosis	No pleocytosis	Unknown	Not reported
Initial ICP	Not reported	Not reported	Not reported	Not reported	Not reported	80 mm	Unknown	Not reported
Species on culture	*neoformans*	Not reported	Not reported	Not reported	*neoformans*	*neoformans*	Unknown	Not reported
Initial MRI changes	Cervical and thoracic	Thoracic	Thoracic	Not reported	Thoracic and lumbar	Thoracic	No MRI	Thoracic

## Data Availability

The data used to support the findings of this study are available from the corresponding author upon request.
